# Novel OA-ICOS Sensor for Real-Time Quantification of Enteric Methane from Ruminants

**DOI:** 10.3390/s26041319

**Published:** 2026-02-18

**Authors:** Yulai Sun, Depu Yao, Jianbo Chen, Guanyu Lin, Jifeng Li, Jianing Wang, Xiaogang Yan

**Affiliations:** 1 Changchun Institute of Optics, Fine Mechanics and Physics, Chinese Academy of Sciences, Changchun 130033, China; sunyulai21@mails.ucas.ac.cn (Y.S.); yaodepu21@mails.ucas.ac.cn (D.Y.); linguanyu1976@163.com (G.L.); lijifeng@ciomp.ac.cn (J.L.); 2 University of Chinese Academy of Sciences, Beijing 101408, China; 3 College of Opto-Electronic Engineering, Changchun University of Science and Technology, Changchun 130022, China; 2023200064@mails.cust.edu.cn; 4 Jilin Academy of Agricultural Sciences, Northeast Agricultural Research Center of China, Jilin 132102, China; yanxiaogang1977@163.com

**Keywords:** greenhouse gas, infrared absorption, off-axis integrated cavity, livestock farming

## Abstract

**Highlights:**

**What are the main findings?**

- A sensing system for measuring methane emissions from ruminants has been developed, enabling real-time detection of methane (CH_4_).
- A spindle-shaped integrating cavity structure is designed to achieve rapid gas concentration replacement and a fast response to changes in gas concentration.

**What are the implications of the main findings?**

- An estimation of daily methane emissions from cattle rumination was conducted, and a correlation analysis was performed between characteristics of methane emission peaks and feeding times.

**Abstract:**

Methane (CH_4_) is a potent greenhouse gas, with livestock rumination being a significant contributor to global emissions. This study developed a real-time monitoring system utilizing Off-Axis Integrated Cavity Output Spectroscopy (OA-ICOS) to simultaneously track rumination behavior and CH_4_ concentrations in cattle breath. By optimizing the off-axis integrated cavity structure and implementing a specialized environmental control system, we enhanced stability and detection accuracy, achieving a rapid 3 s response time to dynamic concentration changes. Laboratory stability tests and Allan deviation analysis demonstrated a minimum detection limit of 0.07 ppm. Continuous field monitoring of Simmental cattle revealed a daily methane production of approximately 311.83 L. The emission rates exhibited a distinct double-peak pattern heavily influenced by feeding schedules. Furthermore, a positive correlation was observed between the time elapsed post feeding and both the frequency and intensity of methane emission peaks. This method enables highly dynamic, stable, long-term monitoring of greenhouse gas emissions from ruminants, providing a robust tool for quantifying emissions and informing scientific feeding practices.

## 1. Introduction

Methane (CH_4_) is the second most potent greenhouse gas contributing to global warming [1]. Agriculture is the primary source of anthropogenic global emissions [2], with livestock systems accounting for approximately 80% of agricultural CH_4_ emissions [3], nearly 90% of which originates from enteric fermentation in ruminants such as cattle [4]. After ingestion, anaerobic microorganisms in the rumen ferment feed, producing CH_4_ as a by-product, which is subsequently released into the atmosphere through eructation during rumination [5]. Accurate quantification of CH_4_ emissions from ruminants like cattle is crucial for understanding the environmental impact of livestock farming and formulating effective mitigation strategies [6,7].

Common methods for greenhouse gas detection include electrochemical sensors, gas chromatography, and various optical techniques [8]. In the context of livestock greenhouse gas monitoring, electrochemical methods [9] offer low accuracy and poor environmental adaptability, making them suitable only for preliminary basic detection. Gas chromatography [10] provides high sensitivity and accurate quantification but involves lengthy analysis cycles, requires sample pretreatment, and is unsuitable for rapid on-site detection. Optical methods are more widely applied in livestock greenhouse gas detection. The respiration chamber method [11] is considered the benchmark for gas detection in animal science due to its high accuracy; however, it confines animals to sealed spaces, which interferes with natural behavior and physiological states, and is both costly and impractical for long-term large-scale monitoring. The GreenFeed system enables automated monitoring of rumination and gas emissions in a non-invasive manner, applicable to free-grazing or housed animals [12]. Nonetheless, it has limited mobility, requires animal training to induce visits for short-term measurements, involves high training costs, offers limited data representativeness, and relies on cloud-based data processing. The SF_6_ tracer technique partially addresses the issue of animal movement, but the complex sampling equipment is prone to damage, laboratory analysis is cumbersome, and temporal resolution is low, preventing real-time continuous monitoring [13]. Non-Dispersive Infrared (NDIR) technology is currently the most widely used gas detection method, offering good stability and strong anti-interference capabilities [14,15]. However, it has a short optical path, relatively high detection limits, and long response times. Cavity Ring-Down Spectroscopy (CRDS) employs high-reflectivity mirrors to form an optical resonator, measuring the ring-down time of laser light in the cavity to calculate gas concentration [16]. It achieves an effective absorption path length of several kilometers and extremely high detection precision, but the equipment is costly, requires precise temperature and humidity control, is sensitive to vibrations, and has a relatively long response time, making it unsuitable for practical measurements in livestock farming environments [17]. Therefore, to detect CH_4_ concentrations emitted by ruminants, it is essential to ensure high detection accuracy while also enabling rapid response to dynamic changes in gas concentration for high-temporal resolution monitoring.

In comparison to the above methods, Off-Axis Integrated Cavity Output Spectroscopy (OA-ICOS) technology is emerging as a research frontier in this field due to its unique advantages [18]. The core breakthrough of this technology lies in its optical resonator structure, which significantly extends the absorption path length, thereby achieving high sensitivity and precision [19]. C Yang et al. used a compact OA-ICOS system to detect CH_4_ by incorporating white noise perturbation, achieving a detection limit of 1.2 ppm and a signal-to-noise ratio of 113.9. However, the system exhibited long-term stability fluctuations of 20 ppm, rendering it unsuitable for prolonged monitoring of cattle rumination [20]. Q He et al. combined OA-ICOS with a PSO-SVM algorithm, achieving a measurement accuracy of 195 ppm and a response time of 20 s [21], yet this still falls short of the requirements for precision and response time in livestock monitoring. Consequently, there is a critical need to develop a CH_4_ sensing system that offers high stability, improved detection accuracy, and shorter response time for monitoring CH_4_ emissions from ruminants in livestock farming.

In summary, this study proposes a CH_4_ concentration detection system for ruminant emissions based on OA-ICOS technology. Through structural design and optimization, an off-axis integrated cavity with a length of 60 mm, a diameter of 22 mm, and an optical path length of 16.67 m was developed, enabling a rapid response time of 3 s to changes in gas concentration. Simulations of the CH_4_ absorption cross-section were conducted, and a DFB laser with a wavelength of 1650.9 nm was selected to effectively avoid interference from other gases. Coupled with a specially designed temperature and pressure control system tailored for this application, the system ensures stable operation over extended periods (on the order of days), making it suitable for long-term deployment and monitoring in actual farming environments. The system was calibrated experimentally under laboratory conditions and subsequently deployed in a practical farming environment to monitor CH_4_ concentrations emitted by Simmental cattle during rumination over an extended period. By analyzing the obtained CH_4_ concentration curves, daily CH_4_ emission amounts and rates from Simmental cattle rumination were estimated, and correlations between post feeding time and characteristics of CH_4_ emission peaks were examined.

## 2. Structure and Design of the CH_4_ Detector

### 2.1. Detection System Design

**Figure 1:**
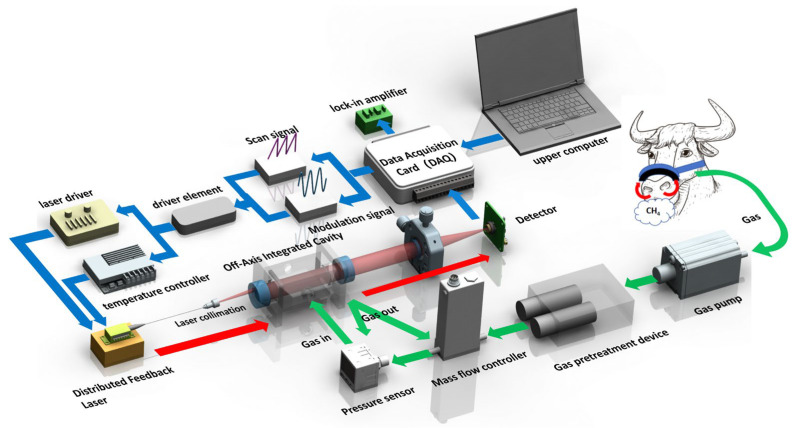
Schematic illustration of the CH_4_ emission detection system.

This study designed a CH_4_ detection system for ruminant animals in livestock farming, as illustrated in Figure 1. The system primarily consists of a tunable diode laser, an off-axis integrated cavity, a photodetector, a drive unit, a data acquisition unit, and a gas path control unit. The tunable diode laser is a DFB laser source with a wavelength of 1650.9 nm. Temperature and current control are applied to the laser to ensure stable operation and precise wavelength tuning. Modulated by TDLAS technology, the laser achieves narrow-linewidth tunable output within a range of several nanometers around the central wavelength [22]. After being collimated by an optical collimator, the laser beam is directed into the integrated cavity. Both ends of the cavity are equipped with mirrors, with a distance of 50 mm between them. Each mirror features a flat outer surface and a concave inner surface with a radius of curvature of 1 m. The inner surface is coated with a high-reflectivity film, providing a reflectance of 99.7%. This design enables an effective absorption path length of 16.67 m after practical assembly and alignment. To ensure operational stability of the cavity structure, the integrated cavity is manufactured from high-strength Invar.

CH_4_ in cattle exhaled gas is the target analyte. A gas pump in the system draws the exhaled gas from a collection device fixed around the cattle’s nose into the subsequent system. After passing through a gas pretreatment system, the dried gas is pumped into the integrated cavity. The target gas absorbs laser light at specific wavelengths, and the remaining light exits the cavity and is focused by a lens with a focal length of 50 mm onto the photosensitive surface of a photodiode. The photodiode converts the light intensity into an electrical signal, which is transmitted to a DAQ unit for signal collection and processing. An environmental control system, including temperature and pressure control, adjusts the system’s temperature and pressure in real time to eliminate the influence of environmental factors on detection.

### 2.2. Analysis of Absorption Spectral Lines

**Figure 2:**
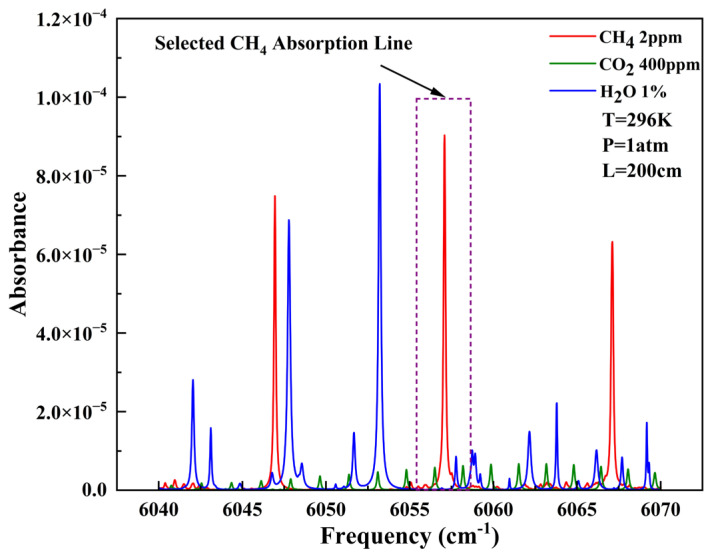
Absorption lines of CH_4_ and interfering gases in cattle exhaled breath at STP.

The practical application scenario of this study involves measuring CH_4_ exhaled by ruminant animals. Based on the HITRAN database [23], the absorption lines of CH_4_ were simulated and analyzed under standard temperature and pressure conditions (296 K, 1 atm). As shown in Figure 2, water vapor (H_2_O) and carbon dioxide (CO_2_), as the main interfering gases in animal exhaled breath, were analyzed in terms of their absorption cross-sections. Comparison revealed that at the wavenumber of 6057.09 cm^−1^ (wavelength of 1650.9 nm), the interference from these two gases on CH_4_ detection is relatively small, with both exhibiting an absorbance approximately one-ninth that of CH_4_. Such interference can be eliminated through subsequent data processing. Moreover, this spectral region meets the requirements for large dynamic range CH_4_ concentration measurements. Therefore, the absorption band of CH_4_ at 6057.09 cm^−1^ (1650.9 nm wavelength) was selected. A DFB laser with a central wavelength of 1650.9 nm was chosen as the light source. The selected tunable laser was modulated with a central current of 62 mA. The temperature of the internal TEC (thermoelectric cooler) module was set to 28 °C. The modulation depth of the sawtooth wave was 20 mA, while that of the sinusoidal modulation wave was 3 mA at a frequency of 2000 Hz.

### 2.3. Design of Gas Pretreatment and Environmental Control

Given that the subjects under investigation are Simmental beef cattle, whose exhaled breath contains a substantial amount of water vapor, and that feeding activities may introduce dust and other particulates into the sampling system, which could be drawn by the vacuum pump into the detection cavity and cause contamination, dust and moisture removal pretreatment of the gas sample is required prior to its introduction into the optical cavity. Considering the high flow rate of the exhaled gas from the cattle and the uncertainty in humidity variation, traditional calcium chloride drying tubes have low adsorption efficiency for water vapor. Moreover, to prevent the formation of gel after calcium chloride adsorbs water vapor, which could block the gas path, regular monitoring of the drying tube is required to replace the adsorbent. Taking into account both dehumidification efficiency and the impact of equipment maintenance on long-term monitoring, this study employs an electronic condenser as the dehumidification device. Its principle is based on the Peltier effect, where cooling is achieved through the cold end, and moisture is condensed and discharged upon reaching the preset temperature. This electronic condenser can control the gas humidity below 20% RH. The dust removal module consists of two stages: the first stage is a micro-fiber filter, which can remove part of the water vapor and impurities. This first-stage dust removal module is placed before the dehumidification module to ensure the proper operation of the dehumidification module. The second-stage dust removal module employs a CEMS (Continuous Emission Monitoring System) secondary filter [24], installed in the gas path after the dehumidification module, which can effectively filter solid impurities of 0.2 µm and larger. Through the above dust and moisture removal pretreatment of the exhaled gas from the cattle, the normal operation of the optical cavity can be ensured.

**Figure 3:**
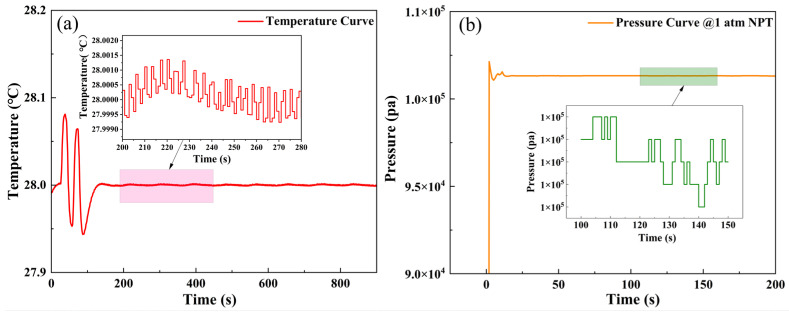
(**a**) Experimental data of temperature control performance. (**b**) Experimental data of pressure CH_4_ control performance.

The performance of the optical cavity in this study is significantly affected by environmental factors, primarily temperature and gas pressure. For temperature control, the optical cavity is placed in a customized EPP material insulation box. The box wall is equipped with a heat dissipation device for internal and external air exchange, consisting of, from inside to out, a cooling fan, a TEC, and a water-cooling refrigeration component. By using PID control for the TEC, precise temperature control is achieved. As shown in Figure 3a, when the temperature is set to 28 °C, the temperature control accuracy reaches 0.002 °C after stabilization. For pressure control, a closed-loop pressure feedback control system is designed. Mass flow meters are installed at the inlet and outlet of the optical cavity to monitor changes in airflow pressure. The pressure control performance is illustrated in Figure 3b. When a sudden pressure change occurs, the pressure response time is less than 20 s, and the pressure fluctuation under steady state is less than 5 Pa. The temperature and pressure control systems ensure the stable and reliable operation of the OA-ICOS detection system in the cattle breath monitoring environment.

### 2.4. Design of a Rapid-Response Cavity

**Figure 4:**
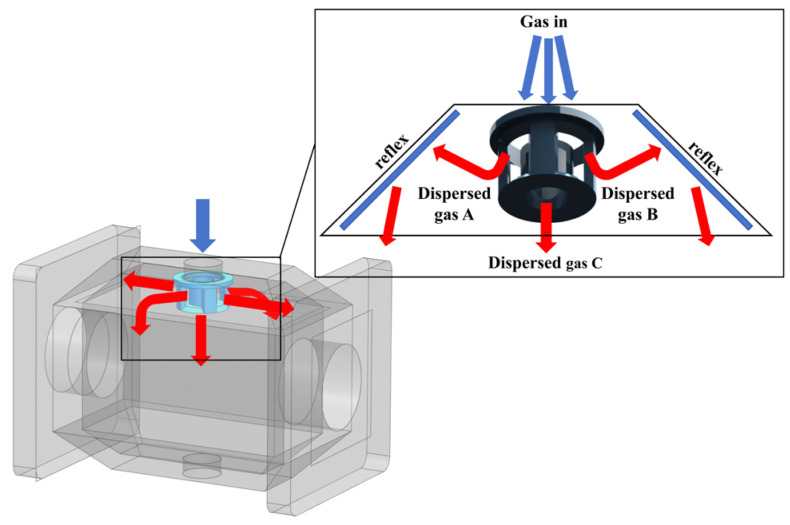
Schematic of the spindle-shaped cavity structure.

The instrument designed in this study is primarily used for dynamically detecting the concentration of in exhaled gas during cattle rumination, which requires the device to have a short response time. The key factor limiting the response time is the duration required for the measured gas to achieve concentration equilibrium as it flows through the cavity. Traditional integrating cavities feature a rectangular cross-section along the axis, with the inlet and outlet typically positioned on the same side of the cross-section. At high flow velocities, vortices tend to form near the right-angle regions of the inner wall, creating gas dead zones that impair gas renewal efficiency. To address this issue, this study proposes a novel spindle-shaped cavity structure along with a matched inlet flow-division grille. To achieve this unique structure while ensuring ease of manufacturing, the integrating cavity is designed as a split structure, with the schematic diagram shown in Figure 4. The structure consists of three components: a main cavity body and two quadrangular pyramid–shaped cavity walls (upper and lower), which are bolted together with sealing elements installed at the interfaces to ensure gas tightness. The inlet flow-division grille is mounted inside the inlet, dispersing the incoming gas flow into two directions: one portion is directed toward the peripheral side, guided by the 45° angled quadrangular pyramid-shaped inner wall, and flows along the wall toward the outlet; the other portion moves directly along a straight channel toward the outlet. This novel structure and flow path significantly reduce gas vortices and dead zones, enabling the measured gas to rapidly achieve concentration equilibrium upon entering the cavity, thereby effectively improving the temporal resolution of the instrument.

To further evaluate the gas concentration refresh rate and equilibrium performance of the newly designed fast-response integrating cavity structure developed in this study, simplified equivalent models were established for both the spindle-shaped cavity structure proposed in this work and a conventional integrating cavity structure, along with their respective gas inlet configurations. Simulations were conducted under an inlet flow rate of 1000 SCCM and a CH_4_ concentration of 0.04% to compare the CH_4_ concentration equilibration within the two types of cavities.

**Figure 5:**
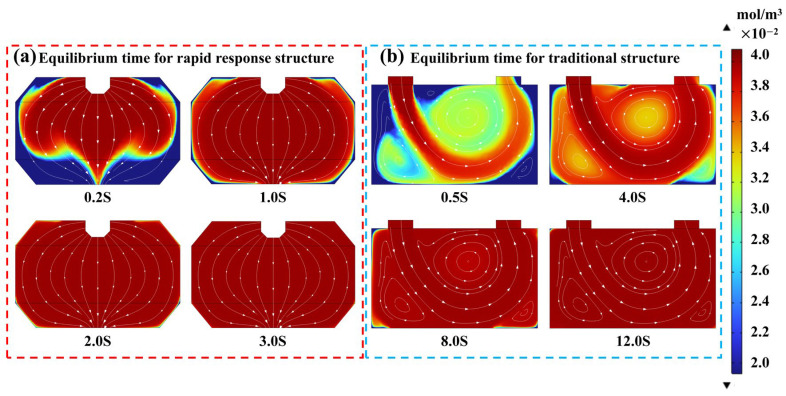
(**a**) Equilibrium time for rapid response structure. (**b**) Equilibrium time for traditional structure.

The simulation results indicate that during gas inflow into the cavity, particularly in the region where the laser beam undergoes multiple reflections, the airflow direction in the spindle-shaped cavity is nearly perpendicular to the laser propagation direction. Owing to the flow-guiding effect of the internal geometric features of the cavity, the airflow does not form vortices, thereby avoiding the creation of concentration dead zones that could adversely affect the gas refresh rate and steady-state equilibrium inside the cavity. Simulation results in Figure 5a indicate that the spindle-shaped cavity structure enables complete gas refreshment within 1.0 s, with the concentration stabilizing after 3.0 s. In contrast, Figure 5b reveals that in the conventional cavity structure, the internal gas refresh requires 8.0 s, and concentration stabilization is achieved only after 12.0 s. The novel cavity structure and its gas inlet/outlet configuration improve the concentration equilibration efficiency by approximately four times, significantly enhancing the temporal resolution of the system. This advancement makes it highly suitable for application in detection scenarios involving rapidly changing gas concentrations.

## 3. Results

### 3.1. Experimental Preparation

In the experiment, a commercial gas mixer SONIMIX-7100 (Switzerland LNI Swissgas, Versoix, Switzerland) was employed to prepare CH_4_ gas at different concentrations. Since N_2_ is the primary constituent of air and O_2_ does not absorb at 1650.9 nm, the collisional broadening effects in N_2_ are nearly identical to those in ambient air, making it a valid proxy for calibration. By mixing 1% concentration CH_4_ standard gas with high-purity nitrogen at different ratios as the gas source, constant-flow CH_4_ gas at various concentrations could be output under the control of two mass flow controllers. The OA-ICOS system was placed in an EPP insulated incubator. A high-precision temperature controller regulated the TEC, combined with water cooling, to achieve high-precision temperature control inside the incubator. Temperature control data were acquired and monitored in real time by an upper computer.

**Figure 6:**
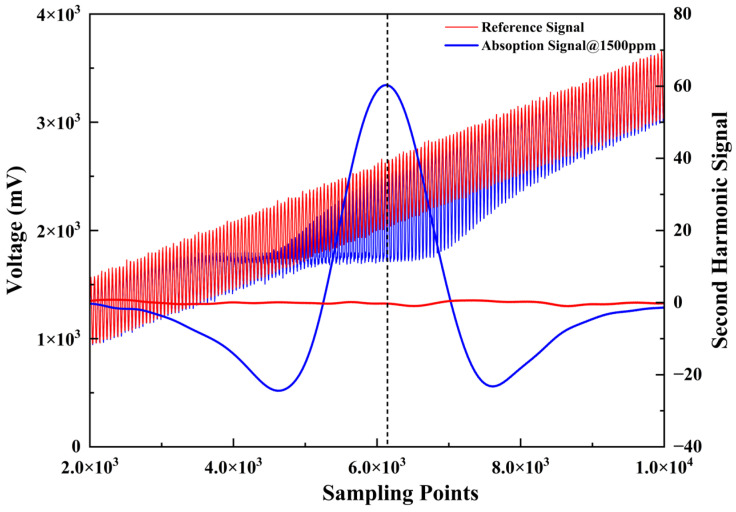
Raw signal and extracted 2f signal upon introduction of N_2_ and 1500 ppm CH_4_.

A sawtooth wave signal and a sinusoidal wave signal were superimposed onto the output of the DFB laser, causing the central wavelength of the laser to scan across the CH_4_ absorption peak at 1650.9 nm. As shown in Figure 6, after demodulating the voltage signal obtained by the detector, the red curve represents the original reference signal obtained by introducing N_2_, and the blue curve represents the original signal after CH_4_ absorption at 1500 ppm. The two signals were differentially processed and subjected to lock-in amplification to extract the second harmonic (2f) signal. The peak-to-peak value of the second harmonic signal exhibits a linear relationship with the gas concentration, which can be used to characterize and quantify the gas concentration.

**Figure 7:**
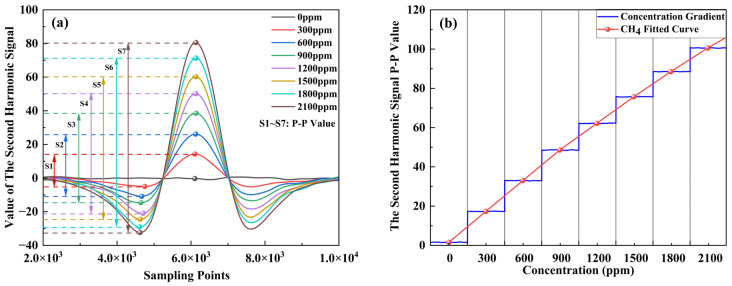
(**a**) Extracted 2f signal for a 300 ppm CH_4_ concentration gradient. (**b**) Calibration of the extracted 2f signal against concentration.

Based on the aforementioned experimental setup, system calibration was performed. The instrument was preheated for 10 min. After the system temperature stabilized, high-purity nitrogen was introduced for 30 min, and the maximum peak-to-peak value of the second harmonic was recorded as the signal value corresponding to 0 ppm concentration. Considering the practical scenario of monitoring CH_4_ concentrations emitted from cattle rumination, the instantaneous CH_4_ concentration peaks during cattle eructation typically range from 300 to over 2000 ppm, a sequence of CH_4_ standard gases with concentration gradients of 300 ppm was prepared by adjusting the mixing ratio of CH_4_ and nitrogen. The peak-to-peak values of the second harmonic signal obtained by the system for each concentration were recorded and extracted, as shown in Figure 7a. For the system calibration, each concentration point was introduced for 20 min, and the average value of the 2f signal from the last 10 min was used as the data representing that concentration (the sampling rate was 1 kHz).

Following the aforementioned experiments, a linear regression analysis was performed on the acquired data. As illustrated in Figure 7b, the peak-to-peak value of the second harmonic signal obtained from the demodulated voltage signal of the detector and the CH_4_ concentration conform to a regression model. Although the Beer–Lambert law predicts a linear relationship at low absorbances, a slight non-linearity was observed in the 2f signal response due to the wide dynamic range (0–2100 ppm) and the long effective optical path length. To compensate for this and minimize measurement error at higher concentrations, a quadratic regression model was employed. A quadratic fit was performed using the acquired second harmonic peak-to-peak values as the independent variable and the set concentration gradient as the dependent variable. The resulting fitting equation is y = 0.03807x^2^ + 18.11x − 19.62, achieving a high coefficient of determination (R^2^) of 0.9998.

### 3.2. System Performance

**Figure 8:**
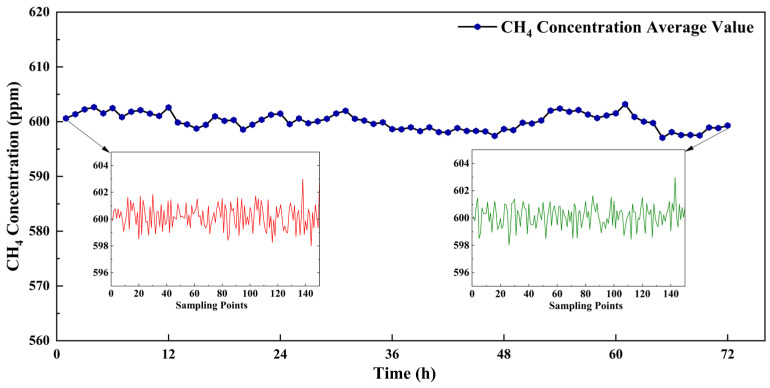
The 72 h detection signal for 600 ppm CH_4_.

Considering the practical application scenario of this study, a 72 h operational stability test was conducted to verify the system’s capability for long-term stable operation. A 600 ppm CH_4_ standard gas was continuously introduced into the instrument via a laboratory gas mixer. Data were collected every 2 s, and hourly averages were calculated to obtain CH_4_ concentration measurements over the 72 h period. As shown in Figure 8, the standard deviation of the detected signal over 72 h for the introduced 600 ppm CH_4_ standard gas was 1.5183 ppm. The standard deviations for the first 5 min and the last 5 min of data were 0.9209 ppm and 0.8167 ppm, respectively. Given that the average CH_4_ concentration emitted during each cattle rumination event exceeds 50 ppm, the observed fluctuation in data deviation was considered negligible compared to the magnitude of CH_4_ exchange during rumination and has almost no impact on concentration retrieval. These results demonstrate that the proposed system is suitable for long-term monitoring of cattle rumination CH_4_ emissions over daily detection cycles. Additionally, for long-term deployment extending beyond several days, the system is programmed to perform an automatic zeroing calibration every 72 h using ambient air or standard nitrogen to eliminate cumulative baseline drift.

**Figure 9:**
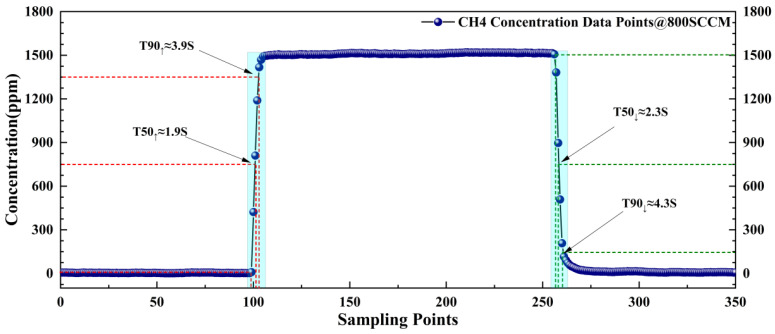
T_50_ and T_90_ response times of the instrument for 0–1500 ppm CH_4_ concentrations.

Secondly, to accurately characterize the capability of the OA-ICOS system in capturing transient changes in CH_4_ concentration within ruminant respiratory flow, this study quantified the instrument’s response time parameters—T_50_ and T_90_ (i.e., the time required to reach 50% and 90% of the target concentration from baseline)—through step concentration tests [25]. Under a concentration change of 1500 ppm, the resulting T_50_ and T_90_ are shown in Figure 9. After alternate rise/fall tests, the system exhibited a rise T_50_ of 1.9 s and T_90_ of 3.9 s, and a fall T_50_ of 2.3 s and T_90_ of 4.3 s. These results demonstrate that the system can fully resolve the CH_4_ concentration profile emitted during cattle rumination within the duration of a single ruminant breath event (>30 s). Combined with the cavity concentration refresh rate and equilibration time derived in the previous chapter, this confirms that the system meets the timeliness requirements for dynamic emission measurements.

Allan variance is an important method for quantifying the stability and detection limit of the OA-ICOS system, as it can be used to analyze and determine the noise characteristics and minimum detectable concentration of the system [26]. During the experiment, N_2_ was purged into the cavity at a constant flow rate using a gas mixer. When the detection signal stabilized, long-term data acquisition was performed to calculate the Allan variance at different integration times.

**Figure 10:**
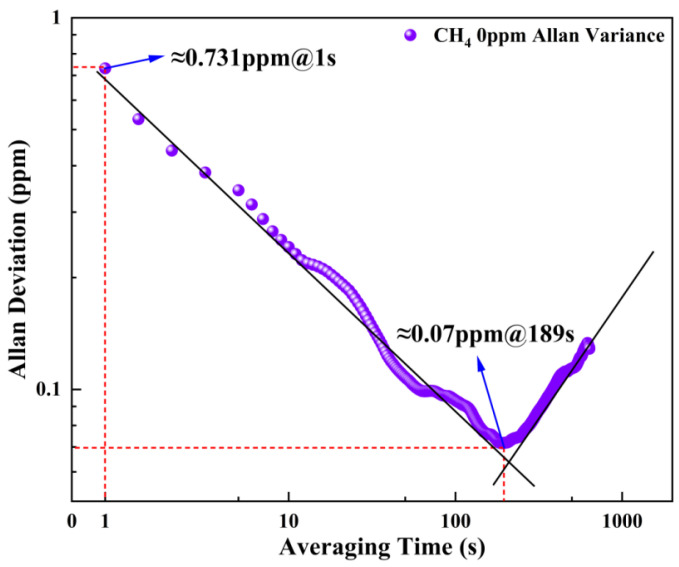
Allan variance and the limit of detection.

In the CH_4_ concentration detection using this system, as shown in Figure 10, the detection limit at 1 s is approximately 0.731 ppm. As the integration time increases to 189 s, the minimum detection limit can reach 0.07 ppm. In practical applications of the system, appropriate adjustment of the integration time enables flexible adaptation to different detection environments and performance requirements.

### 3.3. Monitoring and Analysis of CH_4_ Emissions from Rumination

In this study, a gas sampling system with an inlet diameter of 6 mm was deployed in front of the cattle nostrils to collect exhaled breath from the animals, using a vacuum pump operating at a flow rate of 5 L/min. To account for the gas dilution effect during sampling, a laboratory simulation was conducted. A pressure-reducing valve was used to regulate the outflow velocity of standard gas from a cylinder, and the sampling inlet was positioned 2 mm away from the gas outlet. The collection efficiency of the sampling device was calculated by comparing the measured concentration signals with the known concentration of the standard gas released from the cylinder. This efficiency was then used to correct the raw concentration data. Based on the calibrated concentrations, the total exhaled CH_4_ volume and emission rate from the cattle were estimated. The actual concentration of CH_4_ in the exhaled breath was calculated using Formula (1):

(1) $$C_{e}\;=\;\frac{C_{m}-(1-\alpha )\cdot C_{b}}{\alpha }$$

where the measured concentration is denoted as *C_m_* (ppm), the background CH_4_ concentration is *C_b_* ≈ 1.8 ppm, and the capture efficiency of the cattle exhaled gas by the sampling system is *α* = 0.4. By measuring and subtracting *C_b_* in this equation, the constant interference from ambient methane in the cattle barn environment is effectively eliminated, ensuring that the calculated *C_e_* reflects only the methane contributed by the cattle’s exhalation. Based on the calibrated concentration results and the flow characteristics of the system, the total 24 h CH_4_ emission from cattle can be estimated using Formula (2) below:

(2) $$V_{\mathrm{total}}\;=\;K\cdot \frac{\alpha \cdot Q_{p}\cdot \tau }{10^{6}}\cdot \sum_{i=1}^{N}C_{e,i}$$

Among these parameters, *Q_p_* represents the pump speed conversion value: 83.33 mL/s, *τ* is the sampling interval, *N* is the number of sampling points, and the constant *K* is the CH_4_ mass-to-volume conversion factor: 1.4 (L/g).

Based on the derived 24 h CH_4_ concentration curve during cattle rumination, in conjunction with the estimated total CH_4_ emission, the average CH_4_ emission rate *R_v_* (L/h) and the instantaneous emission rate *R_i_* (L/h) can be calculated using the following equations:

(3) $$\bar{R_{v}}\;=\;\frac{V_{\mathrm{total}}}{24}$$

(4) $$R_{i}\;=\;\frac{\alpha \cdot Q_{p}\cdot C_{e,i}\cdot K}{3600}$$

**Figure 11:**
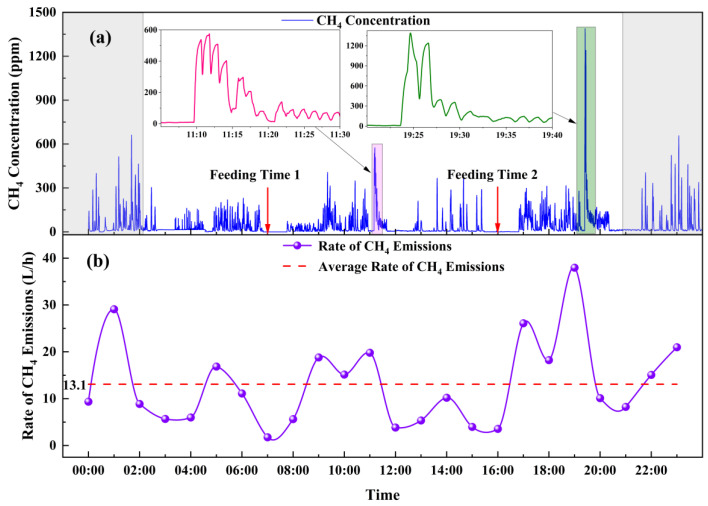
(**a**) CH_4_ emission concentration curve from one subject (Simmental cattle) over a 24 h period. (**b**) CH_4_ emission rate from one subject over a 24 h period.

Based on the aforementioned calculations, the real-time monitoring system designed in this study successfully captured the dynamic changes in rumination behavior and CH_4_ emission concentrations from six Simmental cattle. Figure 11a shows the CH_4_ emission concentration curve detected over 24 h for one of the test subjects—a middle-aged Simmental cattle (22 months old).

As illustrated in the figure, during rumination, the animal exhibited multiple eructation events to release CH_4_ gas, with each spike in the curve representing the CH_4_ concentration captured by the system during eructation. Over the 24 h measurement period, feeding was conducted at 6:00 AM and 16:00 PM, respectively. The feed consisted of a mixture of roughage and concentrate [27]. The roughage included powdered Leymus chinensis and corn straw (4% ≤ CP ≤ 5%), while the concentrate was composed of soybean oil, soybean DDGS (CP > 30%), wheat bran, sodium bicarbonate, salt, limestone, and premix. Using the aforementioned formula, the total CH_4_ emissions from this animal were estimated to be approximately 311.83 L per day (at 25 °C and 1 atm).

Data analysis revealed that, compared to resting periods, CH_4_ emission concentration signals exhibited more frequent and intensive peaks approximately 1.5 h after feeding. This was characterized by a significant increase in CH_4_ concentration accompanied by high-intensity emission peaks, which exhibited a unique rhythmic pattern. In terms of external physiological manifestations, at the onset of rumination, the cattle slightly extended their necks followed by a brief swallowing movement in the reverse direction of normal deglutition (regurgitation). Through reverse peristalsis of the esophagus, the bolus was transported from the rumen back to the mouth. The cattle then continuously chewed the regurgitated bolus with a rhythmic jaw movement, typically at a rate of about 50 times per minute. Monitoring data indicated that the first peak occurred at 11:12:55 AM, with a concentration of 509.83 ppm (corrected to 1435.06 ppm), and the second peak appeared at 19:24:34 PM, with a concentration of 1388.34 ppm (corrected to 3468.07 ppm). This observation aligns with the established mechanism that rumen fermentation activity in ruminants significantly intensifies after feeding, leading to an increase in CH_4_ production. Notably, following high-intensity CH_4_ emission peaks, a diminishing rumination behavior lasting about 30 min was observed, until obvious eructation activities ceased. This is primarily because repeated chewing results in an overly softened rumen content, which reduces stimulation to the rumen and decreases neural afferent impulses, thereby insufficiently triggering effective regurgitation reflexes and gradually halting rumination [28].

Based on the CH_4_ emission concentration curve, the fitted 24 h CH_4_ emission rate is shown in Figure 11b. The intraday emission rate exhibited significant fluctuations: the minimum value occurred around 7:00 AM, one hour after morning feeding, at 1.8 L/h; the maximum value appeared around 19:00 PM, reaching 38.0 L/h. The calculated average CH_4_ emission rate over 24 h was 13.1 L/h. Further analysis revealed that the emission rate curve within six hours after feeding displayed a distinctive bimodal pattern, reflecting the physiological mechanism of rumination—regurgitation of coarse rumen contents back to the mouth (increased emission rate), followed by chewing mixed with saliva and re-swallowing (relatively decreased emission rate). The finer bolus, with increased surface area, promotes further microbial fermentation, leading to a significant rise in CH_4_ emission rate [29]. The diurnal variation trend and emission peak patterns of CH_4_ emissions during rumination observed in this study are consistent with previously reported daily CH_4_ emission data from cattle obtained using automated head chamber systems by Wang Rong et al. [30]. However, with higher temporal resolution, this study accurately captured rapid dynamic changes in CH_4_ emissions during rumination. It not only effectively identified higher CH_4_ emission peaks during rumination but also detected frequent, lower-concentration CH_4_ emission peaks. Additionally, gradually weakening minor emission peaks were observed near the end of rumination, providing more detailed experimental evidence for in-depth analysis of the physiological regulatory mechanisms of CH_4_ emissions in ruminants.

**Figure 12:**
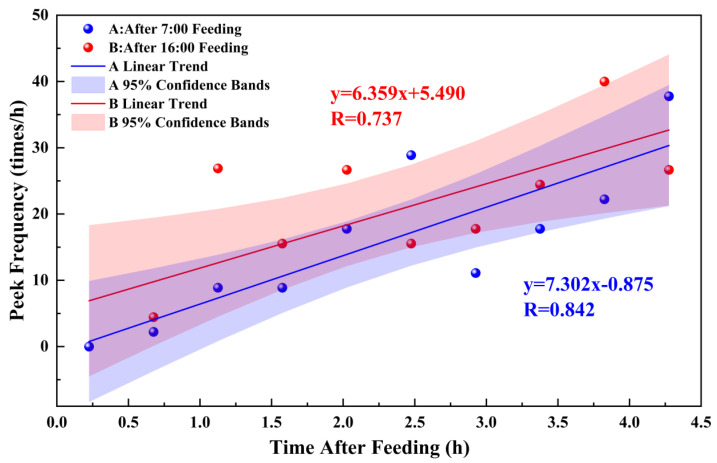
Correlation between the frequency of CH_4_ emission peaks and time after feeding.

Within a certain period after feeding Simmental beef cattle, corresponding changes in the frequency and height of CH_4_ emission peaks were observed over time post feeding, as shown in Figure 12. Within 4.5 h after feeding, the frequency of CH_4_ emission peaks (Y_f_) gradually increased with time (X_f_). Specifically, after morning feeding at 06:00, the frequency of CH_4_ emission peaks was described by Y_f1_ = 7.302X_f_ − 0.875 (R = 0.842, *p* < 0.01), while after afternoon feeding at 16:00, it followed Y_f2_ = 6.359X_f_ + 5.490 (R = 0.737, *p* < 0.01).

**Figure 13:**
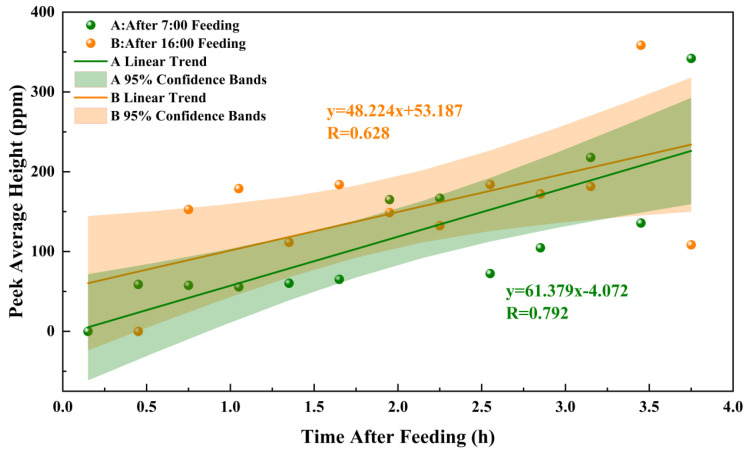
Correlation between CH_4_ emission peak height and time after feeding.

The CH_4_ emission peak height also exhibits certain correlative changes with the duration of time after feeding. Within 4 h after feeding, the regression fit of CH_4_ emission peak height (Y_H_) over time (X_f_) is shown in Figure 13. Specifically, after morning feeding at 06:00, the CH_4_ emission peak height is described by Y_H1_ = 61.379X_f_ − 4.072 (R = 0.792, *p* < 0.01), while after afternoon feeding at 16:00, it is given by Y_H2_ = 48.224X_f_ + 53.187 (R = 0.628, *p* < 0.01).

These results demonstrate that within a certain period after feeding, CH_4_ emissions during digestion and rumination in cattle follow a specific pattern. The characteristics of CH_4_ emission peaks during rumination are closely related to the time after feeding. As time progresses after feeding, the frequency of rumination gradually increases, and the intensity of CH_4_ emissions per rumination event also exhibits an increasing trend.

From the perspective of bovine physiological characteristics, this phenomenon is closely associated with the dynamic process of ruminal digestion and metabolism. In the initial stage of feeding, the ingested feed particles primarily undergo initial soaking and mixing in the rumen. At this stage, functional microorganisms such as fiber-degrading bacteria have not yet entered a highly efficient metabolic phase. The production of volatile fatty acids (VFAs) in the rumen remains relatively low, and the metabolic substrates (e.g., H_2_ and CO_2_) required by methanogens are insufficient, resulting in a slower initiation of rumination and weaker CH_4_ emission intensity [31]. As time elapses after feeding, the feed particles in the rumen gradually become finer due to mechanical mixing and microbial enzymatic hydrolysis. The degradation efficiency of fibrous substrates significantly improves, leading to the accumulation of intermediate metabolites such as H_2_. This provides ample substrates for CH_4_ synthesis by methanogens. Simultaneously, the continuous accumulation of CH_4_ in the rumen enhances the distension stimulus on the rumen wall, which promotes the initiation and increased frequency of rumination through neuroreflex mechanisms. Coupled with efficient substrate metabolism by methanogens, the intensity of CH_4_ emissions per rumination event gradually increases over time.

The above findings reveal the synergistic dynamic relationship among ruminal microbial metabolism, rumination behavior regulation, and CH_4_ emissions in cattle after feeding. This provides a basis for a deeper understanding of the physiological regulatory mechanisms of CH_4_ emissions in ruminants and offers scientific references for strategies such as optimizing feeding schedules and modulating the rumen microbial community to reduce CH_4_ emissions from ruminants.

## 4. Discussion

Compared to previously reported systems such as induced head-chamber measurement and respiration chamber techniques, our study developed a gas detection system capable of rapid response and data processing targeted at CH_4_ emissions from ruminating behavior. The system is relatively simple to operate, causes minimal disturbance to the subjects, and imposes fewer spatial constraints. Experimental results captured the daily variation curve of CH_4_ emission concentration in Simmental cattle. Based on the collected data, the total daily CH_4_ emission and the variation in emission rates were estimated. The estimated results are consistent in order of magnitude with previously reported CH_4_ emission values. Additionally, a positive correlation was observed between cattle ruminating behavior-related CH_4_ emissions and feeding time. Quantifying CH_4_ emissions can provide a scientific basis for formulating targeted emission reduction policies, reveal the patterns of material and energy exchange between ruminants and the environment, offer data support for understanding the carbon cycle equilibrium mechanism in grassland ecosystems, and guide the development and application of emission reduction technologies such as feed formula improvement and innovative breeding models—thereby promoting green and sustainable development of the livestock industry.

However, the current testing system still requires specific optimizations to enhance its robustness in complex field environments. First, regarding the gas sampling efficiency, to minimize the dilution effect and the impact on the animal’s normal behavior, we plan to optimize the aerodynamic design of the sampling inlet. Second, to mitigate the interference of environmental fluctuations, we will implement a multi-sensor fusion compensation strategy. We plan to integrate high-precision anemometers and temperature–humidity sensors at the sampling point to acquire real-time environmental data. This data will be utilized to further optimize the system’s compensation algorithms, thereby improving the measurement accuracy in open-field conditions.

## 5. Conclusions

In this study, an OA-ICOS system was established for the analysis of gases emitted during ruminant behavior, enabling high-precision and rapid real-time monitoring of CH_4_ concentration in animals during rumination. The system is capable of responding quickly to dynamic changes in gas concentration, providing accurate and representative data on CH_4_ emissions from dairy cattle during normal activities. The estimated total daily CH_4_ emissions align well with values reported in previous studies, and correlation analysis revealed significant relationships between CH_4_ emission characteristics and the behavior as well as feeding times of Simmental cattle. This system offers a robust tool for quantifying CH_4_ emissions from ruminants and lays the groundwork for further research on reducing greenhouse gas emissions in livestock farming. Future improvements to the system and expansion of the research scope will contribute to a more comprehensive understanding of cattle CH_4_ emissions and the development of more effective mitigation strategies.

## Data Availability

Data are contained within the article.

## Abbreviations

The following abbreviations are used in this manuscript:

OA-ICOS	Off-Axis Integrated Cavity Output Spectroscopy
NDIR	Non-Dispersive Infrared
CRDS	Cavity Ring-Down Spectroscopy
DFB	Distributed Feedback Laser
TDLAS	Tunable Diode Laser Absorption Spectroscopy
DAQ	Data Acquisition
TEC	Thermoelectric cooler
CEMS	Continuous Emission Monitoring System
EPP	Expanded Polypropylene
DDGS	Distillers Dried Grains with Solubles
CP	Crude Protein
VFAs	Volatile Fatty Acids
